# Ginseng adjuvant therapy on COVID-19: A protocol for systematic review and meta-analysis

**DOI:** 10.1097/MD.0000000000027586

**Published:** 2021-10-29

**Authors:** Hang Shi, Yawen Xia, Renjun Gu, Shuang Yu

**Affiliations:** a The Key Laboratory of Bio-Medical Diagnostics, Suzhou Institute of Biomedical Engineering and Technology (SIBET), Chinese Academy of Sciences, Suzhou, China; b Suzhou Institute of Biomedical Engineering and Technology, Chinese Academy of Sciences, No. 88 Keling Road, Suzhou New District, Suzhou, China; c Nanjing University of Chinese Medicine, Nanjing, Jiangsu Province, China; d Jiangsu Key Laboratory for Pharmacology and Safety Evaluation of Chinese Materia Medica, School of Pharmacy, Nanjing University of Chinese Medicine, Nanjing, China; e Jiangsu Provincial Second Chinese Medicine Hospital, The Second Affiliated Hospital of Nanjing University of Chinese Medicine, Nanjing, China.

**Keywords:** COVID-19, ginseng, meta-analysis, systematic review

## Abstract

**Background:**

Corona virus disease 2019 (COVID-19) is spreading fast and it brings great pressure to the social economy. Many reports revealed that ginseng can develop immunity for respiratory disease, but there is no evidence to prove its effects on COVID-19. This protocol of systematic review and meta-analysis will clarify the safety and effectiveness of ginseng adjuvant therapy on COVID-19 patients.

**Methods:**

Different databases (Web of Science, Cochrane Library, PubMed, Chinese Biomedical Literature Database, Chinese National Knowledge Infrastructure, Chinese Scientific Journal Database, Wan fang Database, ClinicalTrials, World Health Organization Trials, and Chinese Clinical Trial Registry) will be retrieved to search related articles according to pre-defined inclusion and exclusion criteria. Clinical recovery time and effective rates will be assessed as the primary outcomes and any changes of patient's condition will be considered as the secondary outcomes. Subgroup analysis and sensitivity analysis will be conducted to explore sources of heterogeneity. Endnote X9.3 will be used to manage data screening. The statistical analysis will be completed by RevMan5.3 and Stata/SE 15.1 software.

**Results:**

This study will assess the effects and safety for ginseng adjuvant therapy on COVID-19 patients.

**Conclusion:**

The discussion will be considered to determine whether sufficient evidence exists to prove the effects of ginseng adjuvant therapy for COVID-19 patients.

**Systematic review registration:**

PROSPERO (ID: CRD42021277843)

## 1 Introduction

Severe acute respiratory syndrome coronavirus 2 has caused a pandemic of respiratory and cardiovascular diseases, known as corona virus disease 2019 (COVID-19).^[1]^ It mainly causes fever, fatigue, cough, shortness of breath, etc.^[2]^ It has seriously affected the health of society.^[3]^ The World Health Organization endorses supportive care due to lacking of specific drugs.^[4]^ Complementary and alternative therapies are widely used for respiratory disease, especially COVID-19.^[5–7]^ Traditional Chinese medicine has been applied to the treatment of COVID-19 and achieved good effects.^[8]^

Ginseng has been used for more than 2000 years as a traditional tonic medicine. Ginseng is usually used for tonifying Qi.^[9]^ It contains a lot of pharmacologically active ingredients and are often used as tonic in neurasthenia^[10]^ and psychosis, cardiovascular system diseases,^[11]^ and diabetes.^[12]^ It has been proven that herbs with Qi-tonifying character are involved in improving the defence capacity of immune system.^[13,14]^

However, there is insufficient evidence to show the effectiveness and reliability of using ginseng on COV19-19 patient. Therefore, we will conduct a meta-analysis and systematic review to evaluate whether ginseng can be implemented as adjuvant therapy on COVID-19 patients.

## 2 Methods

### 2.1 Review design

This protocol was registered in PROSPERO (CRD42021277843). It refers to the guide book of Preferred Reporting Items for Systematic Reviews and Meta-Analyses Protocols (PRISMA-P).^[15]^ This meta-analysis will be conducted to assess the efficacy and safety of using ginseng as adjuvant therapy for COVID-19 patients.

### 2.2 Search strategy

The following databases will be retrieval by three independent reviewers (HS, YX, and RG): Web of Science, Embase, Cochrane Library, PubMed, Chinese Biomedical Literature Database, Chinese National Knowledge Infrastructure, Chinese Scientific Journal Database, and Wanfang Database, ClinicalTrials, World Health Organization Trials, Chinese Clinical Trial Registry. Languages will be limited to English and Chinese. The publication dates will to limited until November 15, 2021.

**PubMed search strategy is as follows:**

#1. Search “COVID-19” [Mesh]
#2. Search (((((((((((((((2019 novel coronavirus infection) OR COVID19) OR coronavirus disease 2019) OR coronavirus disease-19) OR 2019-nCoV disease) OR 2019 novel coronavirus disease) OR 2019-nCoV infection) OR Wuhan coronavirus) OR Wuhan seafood market pneumonia virus) OR COVID19 virus) OR COVID-19 virus) OR coronavirus disease 2019 virus) OR SARS-CoV-2) OR SARS2) OR 2019-nCoV) OR 2019 novel coronavirus
#3. Search #1 OR #2
#4. Search “ Ginseng ”[Mesh]
#5. Search ((((((((((((((Ninjin) AND (Ninjins)) AND (Renshen)) AND (Renshens)) AND (Shinseng)) AND (Shinsengs)) AND (Jen Shen)) AND (Jen Shens)) AND (Shen, Jen)) AND (Ginseng)) AND (Ginsengs)) AND (Schinseng)) AND (Schinsengs)) AND (Korean Red Ginseng)) AND (Ginseng, Korean Red)
#6. Search #4 OR #5
#7. Search # 3 AND #6

### 2.3 Study selection

#### 2.3.1 Type of study

Randomized controlled trials will also be considered in this meta-analysis. If it doesn’t mention the method of randomization, we will try to contact the authors for more information.

#### 2.3.2 Participants

This study will include patients diagnosed with COVID-19.^[16]^ The patient's characteristic will be recorded, such as age, sex or ethnic origin.

#### 2.3.3 Type of interventions

We will include the literatures that use ginseng as a main variable. Combination therapy involving ginseng and other interventions will be compared with the intervention alone.

#### 2.3.4 Type of comparators

We will include no-treatment, regular treatment or placebo in the control group.

#### 2.3.5 Primary outcomes

Clinical recovery time and effective rate will be considered as the main outcomes. The clinical recovery time is defined as the time from initiation of study treatment (active or placebo) until normalisation of fever, respiratory rate, and oxygen saturation, and alleviation of cough, sustained for at least 72 hours. If the literatures describe the effectiveness without same standards. We will contact author of that literature for further information.

#### 2.3.6 Secondary outcomes

1. Time to COVID-19 RT-PCR negative in upper respiratory tract specimen.
2. Time to cough reported as mild or absent.
3. Time to dyspnea reported as mild or absent.
4. Frequency of requirement for supplemental oxygen or noninvasive ventilation.
5. Frequency of respiratory progression.
6. Severe case incidence.
7. Proportion of re-hospitalization or admission to ICU.
8. All-cause mortality.
9. Frequency of serious adverse events.

If a new suitable form is found in the literature search, it will be taken into consideration.

#### 2.3.7 Exclusion criteria

1. Literatures published repeatedly by the same author or with duplicate data;
2. Full data cannot be obtained after contacting with author; and
3. Literatures with less than 10 samples in experimental group or control group.

#### 2.3.8 Data extraction

**Figure 1:**
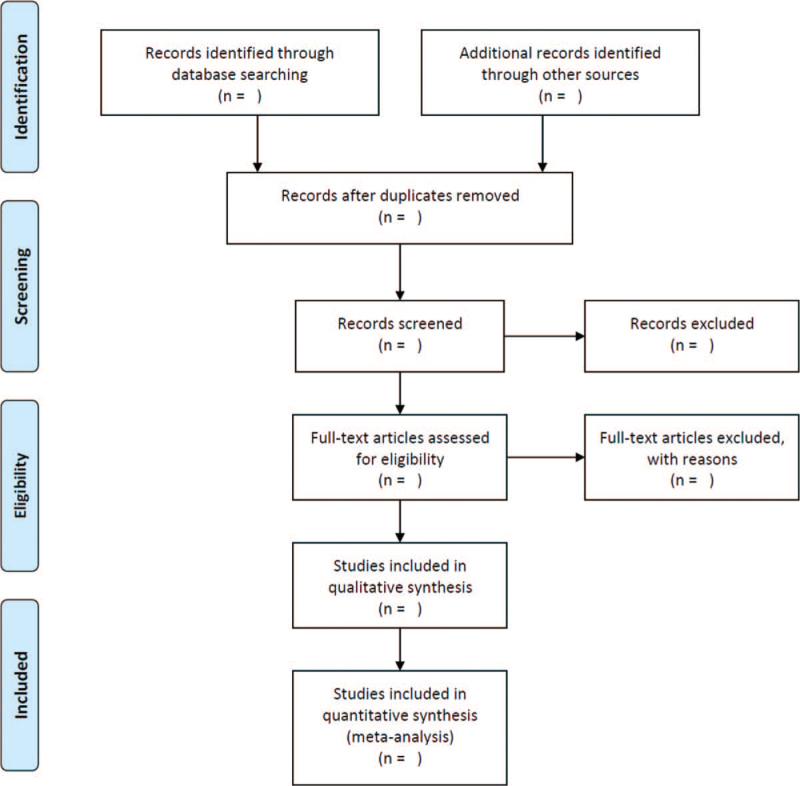
PRISMA flow diagram of the study process. PRISMA = Preferred Reporting Items for Systematic review and Meta-analysis.

Endnote X9.3 (Clarivate Analytics) will be used to manage data screening. The statistical analysis will be completed by RevMan5.3 (Cochrane). Three review authors (HS, YX, and RG) will include articles through title and abstract independently. A third expert (SY) will take it into discussion when having any disagreements. If the missing data cannot be obtained, then the study will be excluded from the analysis. We will use PRISMA flow chart to present the process of study selection (Fig. 1).

#### 2.3.9 Risk of bias assessment

According to Cochrane Collaboration's tool for assessing risk of bias,^[17]^ the quality of evidence of the articles included in this review will be assessed by one reviewer, and confirmed by a second one. We will discuss when difference exists in risk of bias assessment. Random sequence generation, assignment concealment, blinding of participants and personnel, blinding of outcome assessment, incomplete outcome data, selective reporting, and other bias will be assessed to evaluate the bias risks. Afterwards, we will provide a chart using green, yellow, red colors and “+”, “−”, “?” to indicate “low risk bias”, “high risk bias”, and “unclear”.

### 2.4 Statistical analysis

#### 2.4.1 Data synthesis

First, we will combine the collected data according to characteristics of eligible trials. For efficiency, we will use fixed effect model to express risk ratio with 95% confidence intervals. On the other hand, the random effect model will be used for continuous outcomes due to clinical heterogeneity. Weighted mean differences or standardized mean differences will be calculated with 95% confidence intervals to assess whether data is statistical significance. Secondly, we will use *P* value and I^2^ value to test heterogeneity between trial results. If the I^2^ > 50%, it means that there is heterogeneity among the consolidated data. The statistical package (RevMan 5.3) will be used for data analysis and forest plots in data presentation. When we find obvious heterogeneity among the combined data, we will explore the source of heterogeneity with subgroup analysis, sensitivity analysis and evaluate publication bias.

#### 2.4.2 Subgroup analysis

Subgroup analysis will be carried out to assess the influence of ginseng on COVID-19 patients with different characteristics (gender, age, basic diseases, etc). It will analyze potential sources of heterogeneity.

#### 2.4.3 Sensitivity analysis

Sensitivity analysis is to assess the bias factors by eliminating each study one at a time. It will be conducted to measure the stability and reliability of the meta-analysis result, and search for the causation of heterogeneity.^[18]^

#### 2.4.4 Publication bias

Publication bias is commonly associated with inflated treatment effect which lowers the certainty of decision makers about the evidence. It will be evaluated by using funnel chart and completed by RevMan 5.3 software. When we have adequate samples, funnel chart will be used to assess bias in small samples.^[19]^

#### 2.4.5 Quality of evidence

According to grading quality of evidence and strength of recommendations,^[20]^ GRADE system will be used to assess quality of literature with high, moderate, low, or very low level.

## 3 Discussion

Now the ongoing pandemic of COVID-19 brings an unprecedented challenge to public health in the 21st century.^[21]^ COVID-19 may lead to changes in the respiratory system,^[22]^ digestive system,^[23]^ cardiovascular system,^[24]^ etc. The COVID-19 pandemic has brought great much pressure to society^[25]^ and has no effective medical cure.

Traditional Chinese medicine has a long history in China and other Asian countries, and plays an indispensable role in the prevention and treatment of severe epidemic diseases.^[26]^ It was proven effective according to different researches.^[27–29]^ At the meantime, ginseng and its extract have been proved to have antiviral function.^[30]^ If ginseng could help with COVID-19 treatment or enhance respiratory function, it will benefit patients and relieve medical pressure. However, there is still no evidence to verify its effectiveness and safety for COVID-19 patients at present. Therefore, this systematic review and meta-analysis will be conducted to assess the application of ginseng adjuvant therapy on COVID-19 patients and provide references for clinical treatment.

Strengths and limitations will be highlighted during identifying evidence. The data extraction and risk of bias assessment will be completed by three researchers independently. Furthermore, the protocol was registered PROSPERO. It will follow the guideline of Cochrane and PRISMA statement.^[17]^ Limitations will mainly be originated from different clinical condition on COVID-19 patients. It may exist clinical heterogeneity. Funnel plots, subgroup analyses and sensitivity analysis will be conducted to explore the sources of heterogeneities for evaluating better evidence.

## Author contributions

**Conceptualization:** Hang Shi, Yawen Xia, Renjun Gu, Shuang Yu.

**Investigation:** Yawen Xia.

**Methodology:** Hang Shi, Renjun Gu, Shuang Yu.

**Writing – original draft:** Hang Shi, Yawen Xia.

**Writing – review & editing:** Renjun Gu, Shuang Yu.
